# NeuroChip: A Microfluidic Electrophysiological Device for Genetic and Chemical Biology Screening of *Caenorhabditis elegans* Adult and Larvae

**DOI:** 10.1371/journal.pone.0064297

**Published:** 2013-05-22

**Authors:** Chunxiao Hu, James Dillon, James Kearn, Caitriona Murray, Vincent O’Connor, Lindy Holden-Dye, Hywel Morgan

**Affiliations:** 1 Hybrid Biodevices Group, Electronics and Computer Science, University of Southampton, Southampton, United Kingdom; 2 Centre for Biological Sciences, Institute for Life Sciences, University of Southampton, Southampton, United Kingdom; Inserm U869, France

## Abstract

Genetic and chemical biology screens of *C. elegans* have been of enormous benefit in providing fundamental insight into neural function and neuroactive drugs. Recently the exploitation of microfluidic devices has added greater power to this experimental approach providing more discrete and higher throughput phenotypic analysis of neural systems. Here we make a significant addition to this repertoire through the design of a semi-automated microfluidic device, NeuroChip, which has been optimised for selecting worms based on the electrophysiological features of the pharyngeal neural network. We demonstrate this device has the capability to sort mutant from wild-type worms based on high definition extracellular electrophysiological recordings. NeuroChip resolves discrete differences in excitatory, inhibitory and neuromodulatory components of the neural network from individual animals. Worms may be fed into the device consecutively from a reservoir and recovered unharmed. It combines microfluidics with integrated electrode recording for sequential trapping, restraining, recording, releasing and recovering of *C. elegans*. Thus mutant worms may be selected, recovered and propagated enabling mutagenesis screens based on an electrophysiological phenotype. Drugs may be rapidly applied during the recording thus permitting compound screening. For toxicology, this analysis can provide a precise description of sub-lethal effects on neural function. The chamber has been modified to accommodate L2 larval stages showing applicability for small size nematodes including parasitic species which otherwise are not tractable to this experimental approach. We also combine NeuroChip with optogenetics for targeted interrogation of the function of the neural circuit. NeuroChip thus adds a new tool for exploitation of *C. elegans* and has applications in neurogenetics, drug discovery and neurotoxicology.

## Introduction

Genetic and chemical biology screens on the nematode *Caenorhabditis elegans* have provided fundamental insight into neural function encompassing key aspects of neurosecretion, synapse formation and regeneration [1]–[3]. These approaches are based on visual observation to delineate phenotypes. Recently microfluidic devices have been designed that facilitate *C. elegans* screens by allowing controlled immobilization [4]–[7], high resolution imaging [5], [6], [8]–[10] and behavioural or developmental observation [11]–[19]. Not only has this enabled high-throughput studies [20]–[24] but it has also delineated new phenotypes by virtue of refined analysis [25].

The ability to screen for *C. elegans* synaptic phenotypes based on an electrophysiological signature would add a new level of refinement to probing synaptic function. Indeed mutations that alter synaptic transmission are not always associated with an obvious morphological or behavioural phenotype [26]. Typically, electrophysiological recordings, particularly in *C. elegans*, are technically challenging and time-consuming and thus not suited to screening approaches. However, the *C. elegans* pharyngeal neural network provides an opportunity to circumvent this. Its activity, the electropharyngeogram (EPG), can be captured by a suction electrode placed over the mouth of the intact worm [27]. This records the electrical signal of the rhythmically pumping pharynx to monitor frequency and duration of the contraction-relaxation cycle. Moreover, fine features of the EPG are synaptically driven events from excitatory and inhibitory neurones and can report on neural signalling and neuromodulatory components of the circuit [27]–[31]. Recently microfluidic devices have been developed which replace the need for manual trapping and conventional microelectrodes [32]–[34] one of which has made progress towards resolving synaptically driven events in the EPG by adopting a new two layer design [34]. Here we report the development of a device based on this principle which we have called ‘NeuroChip’ because it yields high resolution EPG recordings in which the aforementioned synaptic events can be reliably detected in recordings of low level basal activity and during periods of rapid, stimulated activity. It has been optimised for trapping worms in the correct orientation, mimicking the shape of a conventional microelectrode aperture and for rapid drug or chemical application. It also incorporates an integral electrode to facilitate ease of use. We demonstrate selection of mutant from wild-type worms on the basis of their distinctive electrophysiological signature. The device is further configured to allow optogenetic interrogation of the neural network. Taken together these features of NeuroChip endow it with the capability to select discrete electrophysiological synaptic and neuromuscular phenotypes from mixed populations of worms, a property commensurate with it performing as a robust platform for genetic and chemical biology screens with potential to deliver new insight into neural function and to facilitate drug discovery.

## Materials and Methods

### Design of the NeuroChip

**Figure 1:**
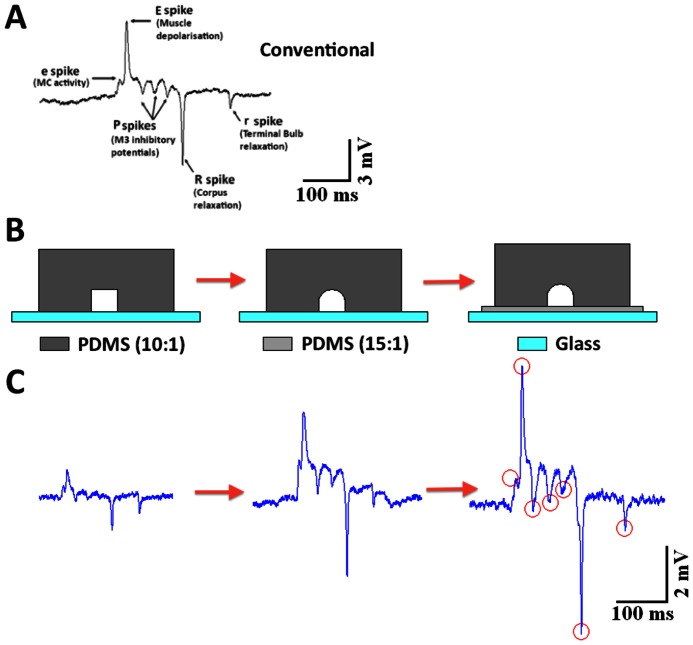
A comparison of different preliminary designs of microfluidic trapping channel and the resulting electropharyngeogram (EPG) signal. **A.** An example of a single pharyngeal pump recording, EPG, obtained using a conventional suction microelectrode placed over the mouth of the worm using a cut head preparation. This waveform depicts three phases: E (e, E), P, and R (R, r) phase which report on the activity of the neural circuit that regulates the worm’s feeding behaviour (see text). **B**. Evolution of the shape of the aperture and **C**. the corresponding EPG signals. The first signal is obtained from a single layer chip and the aperture which traps the worm’s anterior is square, which is similar to the device described by Lockery et al. [33]. The signal in the middle is taken from a single layer chip with semi-circular aperture, which is more similar to the shape of the worm. The last example of an EPG signal is recorded from a two-layer chip with semi-circular shape aperture. The softer base provides a better seal and thus generates a larger amplitude EPG and improved resolution of subcomponents of the waveform reporting neural activity. Each phase and feature of the EPG are of greater magnitude in this last waveform. All the features are marked with red circles.

**Figure 2:**
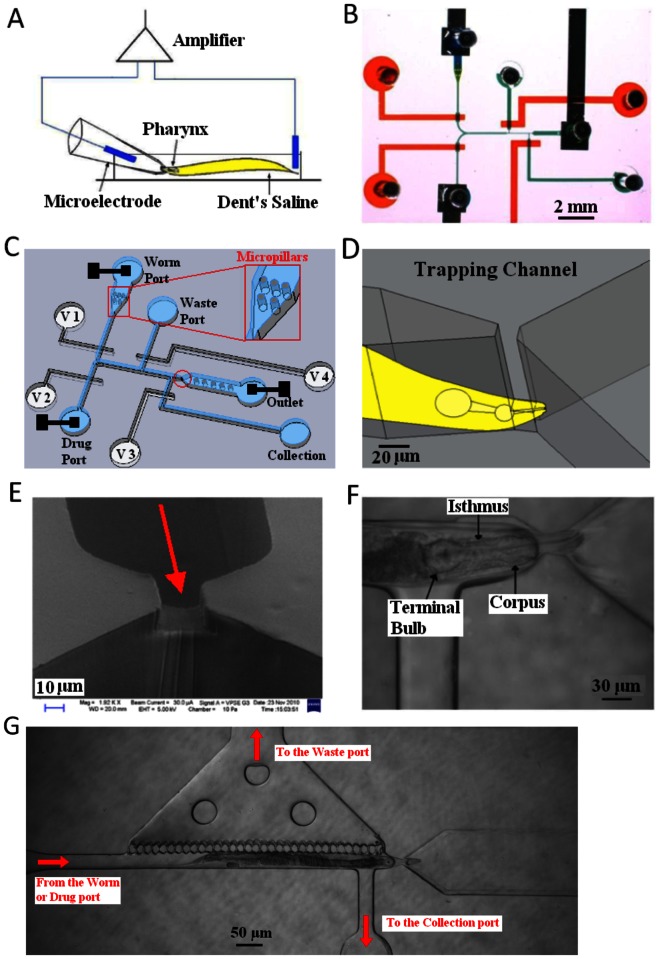
Conventional and microfluidic methods for extracellular electrophysiological recording from the *C.* *elegans* pharyngeal nervous system. **A.** Schematic of conventional microelectrode EPG recording setup. **B.** Photograph of the microfluidic NeuroChip. Channels filled with food dye: Green flow layer; red control layer. **C & D**. Diagram of the NeuroChip. It is a two-layer structure: the blue layer is the microfluidic region processing worms and delivery of drugs. Trapping region (red circled) is magnified in D, showing the trapped worm’s head. The red square is the micro-pillar region which facilitates correct orientation of the worm. The white layer is the pneumatic control layer (V1, V2, V3 and V4 indicate Valve 1, 2, 3 and 4 respectively). The three black squares are the microelectrodes. **E**. SEM image of the trapping region. **F**. Photograph of a worm trapped in the device. The worm is trapped at the front part of its corpus. **G**. Addition of a wide perforated chamber alongside the worm to facilitate rapid drug access (see text for details).

To achieve a high quality EPG signal, a robust and electrically tight seal around the worm’s head is required. In order to design a trapping channel that mimicked the capability of the conventional electrode to extract discrete neural components of the waveform the fabrication of the microfluidic device was optimised over several iterations. The key steps are indicated in Fig. 1B. The incorporation of a soft under-layer and an additional procedure to form semi-cylindrical channels so that the microfluidic chamber has a rounded ‘ceiling’ ultimately permitted the robust capture of EPG signals that were indistinguishable from those obtained using conventional glass microelectrodes (Fig. 1 B, C; Fig. 2 A). Before this optimisation the amplitude and waveform were similar to that reported with the device designed by Lockery et al (Fig. 1C, first recording) [33].

Consistent precise placement of the worm in the trap facilitates reproducibility of the recorded EPG waveforms. The optimum size for trapping a one day old adult hermaphrodite was determined by fabricating a range of different channel dimensions ranging from 9 µm×5 µm×15 µm to 40 µm×12 µm×30 µm (width×height×length). Young adult worms (L4+1 day) were chosen. The design was further modified to capture L2 larvae a distinct advantage over conventional recordings as these are extremely difficult to perform on these small (less than 500 µm), very motile worms.

The device was designed in two layers of PDMS (polydimethylsiloxane). The top layer is the microfluidic layer, which consists of the trapping channel and several bypass channels (green in Fig. 2B). The bottom layer also contains the valve control layer (red in Fig. 2B) with four pneumatic micro-valves [35]–[37]. The bypass channels are 50 µm wider than the dimension of young adult (L4+1 day) *C. elegans* and are used to transport worms and drugs. The device is designed so that worms can be loaded into the channel in solution individually (via a pipette tip inserted into the PMDS) or from a reservoir attached to the chip. Individual worms enter the channel from the inlet marked ‘worm port’ (Fig. 2C). In initial experiments it was found that the worm enters the channel in a random orientation and was only in the correct orientation (i.e. head first, Fig. 2 D, E, F) 42% of the time. It has previously been reported that worms preferentially adopt a head orientation if small pillars are fabricated within such entry channels [10]. These pillars act as obstacles to the worm’s movement towards the trap. When a constant hydrostatic pressure is applied to the inlet port, worms escape through this micropillar array in the appropriate (head-to-tail) orientation. Thus we incorporated micropillars (50 µm in diameter) into the design of the EPG chip (Fig. 2C) and this increased the percentage of worms that entered the channel head first to 71% (of 55 worms tested). The selection of correctly oriented worms was made using valve 3 (Fig. 2C). If a worm enters tail-first, it may be flushed away through the collection outlet by opening this valve.

The device also allows drugs, compounds or chemicals to be washed on and off the worm while it is trapped in the recording channel. This was achieved by an additional drug port (Fig. 2C) used to deliver drugs to the worm. In order to improve drug delivery to the worm in the trapping channel the chamber was modified to allow access of the drug along the entire length of the worm’s body at once. This was done by adding a wide chamber along one side of the trapping channel as shown in Fig. 2G. This chamber was separated from the main body of the worm by a perforated partition punctuated with a row of 10 µm holes. This improved design allowed the drug to rapidly access the length of the worm. Videos S1 and S2 compare dye access to the chamber without and with the perforated partition, respectively. Without the perforated side chamber the access time was 46±5.4 sec, n = 5± s.d., whilst with the perforations the dye access occurred within seconds. Furthermore, this design reduced the time required to wash out the drug.

The device was designed to incorporate integrated electrodes. Thus it circumvents the need for a separate electrode and allows for direct connection to the recording amplifier.

### Device Fabrication

Devices were fabricated using soft lithography [38] using a two-step process. Acetate masks (Micro Lithography Services Ltd, Essex, UK) were used to define large scale features. Masters for casting PDMS were made from SU8-5 (Chestech, Warwickshire, UK). To make the device a thin layer of SU8 was spin-coated on a wafer to make the trapping channel (10 µm thick). Positive resist (AZ40XT, MicroChemicals GmbH, Germany) was used to form the flow channel which is 60 µm high. SU8-50 (Chestech, Warwickshire, UK) was spin-coated on another wafer to provide the pneumatic control layer (80 µm thick). After exposure of the resist, the flow layer master was baked at 130°C for 30 seconds to reflow the resist to form semi-cylindrical flow channels. Before moulding the PDMS surfaces were treated with trichloro (1H, 1H, 2H, 2H-perfluorooctyl) silane 97% vapour (Sigma-Aldrich). The channel was made from PDMS (10 part A: 1 part B) to a thickness of ∼ 5 mm. The control layer was created by spin-coating the PDMS mixture on the control layer master at 900 rpm for 30 seconds, yielding a thickness of 100 µm. After degassing for 30 minutes and curing at 100°C for 1 hour, the PDMS replicas were peeled off the masters. Holes for different ports were punched and cleaned before bonding. After treatment with oxygen plasma (50 W, 30 s), the PDMS flow layer was bonded onto control layer with hand alignment. The same process was used to bond them onto a glass cover slide which acts as the base. Finally, sections of polythene tubing were attached to the chip ports. Four solenoid valves (Clippard minimatic, 3-way normally-closed valve) were used to achieve the external valve control. The inlet is connected to an air compressor and the outlet is connected to the NeuroChip. When an excitation voltage of 6 volts is applied to the solenoid valve, the solenoid pulls the valve into the open state and the compressed air pressurizes the micro valve on chip. An I/O board (Phidgets USB Interface 0/16/16) is connected between the power supply and solenoid valves, playing the role of physical switches, to control the on/off states of the valves automatically. The micro-valves in the bottom layer are driven by software and provide a semi-automatic means of loading a single worm, recording an EPG signal, drug delivery and worm unloading.

The three platinum electrodes were fabricated on the glass substrate, at the entrance of the three fluid ports (Fig. 2B and C). Holes were drilled through the two PDMS layers to expose the electrodes to the solution. These integrated electrodes simplify worm handling and markedly improve the signal to noise ratio (SNR). The EPG signals recorded from this chip have a SNR larger than 8, providing the capability for fine resolution of features in the EPG waveform. All electrodes are connected to a conventional high-input impedance amplifier. The electrodes under the worm port and drug port are connected together to the recording electrode, whilst the electrode in the outlet port is grounded. The NeuroChip is placed in a Faraday cage.

### Operation of the Device

Valves 2, 3 and 4 (V2, V3, and V4 in Fig. 2C) were actuated to close the bypass channels. The worm was pumped by positive pressure (0.3 mBar) into the chip from the worm port by compressed air controlled by the computer. All other ports were at atmospheric pressure. At this small constant positive pressure, the worm was pushed into the trapping region (see Fig. 2E). Electrophysiological signals were then captured and either observed in real time or recorded for *post hoc* analysis. If the worm was in the correct orientation for EPG recording i.e. nose first, then the experiment was continued. If not, the worm was rapidly unloaded from the chip (by closing valve 2 and opening valve 1 and 3). Drugs were applied by closing valve 1 (V1 in Fig. 2C), and opening valve 2 and 4, thus delivering drugs from the drug port. To facilitate access of the drug to the worm, valve 4 (the waste channel) was closed and the drug was allowed to flow into waste port around the worm. The EPG was then recorded in the presence of the drug. Drugs can be washed off the worm by closing valve 2 and opening valve 1. Finally valve 2 and 4 was closed and valve 1 and 3 opened to unload the worm from the device. This worm could be collected after EPG recording e.g. for genotyping by PCR, and the device was then ready to receive the next worm.

### Worm Culture and Sample Preparation

Wild-type *C. elegans* (N2, Bristol) and *eat-4* mutants (strain MT6308, *eat-4*(*ky5*)III and strain MT6318, *eat-4*(*n2474*)III) were cultured according to standard protocols [39]. Experiments were performed on age-synchronized worms, either L2 larvae or L4 plus one day old adults. Dent’s saline compound (composition in mM; D-glucose 10, HEPES 10, NaCl 140, KCl 6, CaCl_2_ 3, MgCl_2_ 1, pH 7.4 with NaOH) was injected into the chip to provide a liquid environment. The *peat-4::ChR2;mRFP* integrated line (described below) was crossed into *eat-4 (ky5)* to generate a strain carrying a visual marker, red fluorescent protein, for the *eat-4* mutant, as follows: Males carrying the integrated transgene were produced by heat-shocking L4 hermaphrodites at 30°C for 6 hours. A population of males was maintained by crossing with L4 hermaphrodites from the same line. Young males carrying the integrated transgene were mated with L4 *eat-4(ky5*) hermaphrodites. In the F1 generation, L4 hermaphrodites expressing mRFP were picked to individual plates and allowed to self. In the F2 generation, L4 hermaphrodites expressing mRFP were picked to individual plates and allowed to self. Their progeny, the F3 generation, were screened for plates where all F3 animals expressed mRFP, i.e., the F2 hermaphrodite was homozygous for the transgene. This screening was carried out before the first F3 progeny reached the adult stage, allowing the F2 animal from such plates to be identified and removed for single worm lysis and genotyping of the *eat-4(ky5)* allele. Genotyping of the *eat-4(ky5),* a 613 bp deletion, was carried out by PCR as described below.

### Conventional Microelectrode EPG Method

Experiments for recording EPGs from intact worms and cut heads were performed as previously described [40].

### Single Worm PCR

This was performed to detect the genomic deletion in the mutant *eat-4(ky5).* Primers used to amplify over the *eat-4(ky-5)* genomic breakpoints were 5′-GCTTGTCAGAAGACAAGTGC-3′ and 5′-CATATGATCCTGTGAATGC-3′. Single worm PCR was performed according to established protocols [41] using optimised cycling parameters and Taq polymerase (Qiagen).

### Data Acquisition and Signal Analysis

The NeuroChip was connected to an AxoClamp 2A amplifier using a x1 headstage (Axon Instruments, USA). Recordings were acquired using a Digidata 1320 interface and Axoscope software (Axon Instruments, USA). Axoscope recordings were made for 5 minutes for the comparison of EPG parameters between conventional and chip recordings, and for 2 minutes for the screening experiments. They were analysed either manually or using AutoEPG [31], as indicated. The EPG parameters that were measured were i) peak to peak amplitude (manually) ii) average frequency (AutoEPG) iii) the duration of a single EPG waveform or ‘pump’ measured as the peak E to peak R time interval (AutoEPG) iv) the interval between consecutive individual pumps measured as the R to E interval (AutoEPG) v) the average number of ‘P’ waves per pump (AutoEPG) vi) the average amplitude of ‘e’ (manually by measuring the perpendicular distance of the peak of the waveform from the baseline of the EPG) vii) the ratio of the amplitude of ‘R’ to ‘E’, as an overall measure of EPG shape (AutoEPG). Where indicated in the results, low frequency drift was removed by Clampfit 9.0 (Axon Instruments) with a Highpass Bessel filter and 0.5 Hz −30 dB cut-off frequency.

### Optogenetics

A strain of *C. elegans* expressing ChR2 specifically in glutamatergic neurones was generated. The method is based on that previously described for expression of ChR2 in cholinergic neurones [42]. For expression in glutamatergic neurones, *ChR2* was amplified from the plasmid *pmyo-3::ChR2(gf)::yfp* (provided by Alexander Gottschalk). ChR2 was amplified using primers that introduce restriction sites flanking the coding sequence: (from 5′-3′): ChR2-SpeI-F: CTAGAGACTAGTATGGATTATGGAGGCGCCCTG; and ChR2-KpnI-R: ATGGGGTACCttaGGGcACCGCGCCAGCCTCGGCCTC. The R primer was designed to introduce a stop codon at the end of the ChR2 coding sequence and a synonymous substitution that disrupts the KpnI site in the 3′ end of the *ChR2* coding sequence, shown in lowercase in the primer sequence. *mRFP* was amplified with the following primers: (from 5′-3′): mRFP-AgeI-F CTA GAA CCG GTC AAT GGC CTC CTC CGA GGA CG and mRFP-EcoRI-R GCA CTG AAT TCT TAG GCG CCG GTG GAG TGG C. PCR was carried out according to standard procedures and purified PCR products were subcloned into pCR-BluntII-TOPO (Invitrogen). The fidelity of the DNA amplification was verified by sequencing (MWG). *ChR2* and *mRFP* were cut out of the TOPO constructs with SpeI/KpnI and AgeI/EcoRI respectively and sequentially ligated into a Gateway pENTR backbone. XL-10 Gold cells (Stratagene) were transformed with the ligation product according to the manufacturer’s instructions. The entry clone *pENTR::ChR2;mRFP* was recombined with a Gateway destination vector containing the promoter region of *eat-4*, *pDESTpeat-4* (*pDESTpeat-4* contains the *eat-4* promoter region from nucleotide position −6022 to −1), in an LR recombination reaction according to the manufacturer’s instructions (Invitrogen) to produce *peat-4::ChR2;mRFP*. To generate transgenic lines, an injection mix containing 1× injection buffer (final concentration 2% polyethylene glycol, molecular weight 6000–8000, 20 mM potassium phosphate, pH 7.5, 3 mM potassium citrate, pH 7.5), and *peat-4;ChR2;mRFP* (50 ng/µl) was injected into the gonads of wild-type (N2) day old adult *C. elegans*. Each injected worm was transferred to a new plate and incubated at 20°C. F1 animals expressing mRFP were picked to new plates. Lines were established from plates where expression of mRFP persisted in the F2 generation. The extrachromosomal array was integrated into the genome by UV irradiation. 25–30 L4 hermaphrodites carrying the extrachromosomal array, as determined by visual confirmation of mRFP expression, were transferred to three unseeded NGM plates. These plates, with lids removed, were subjected to a UV dose of 300 J/mz in a Stratagene UV crosslinker. UV irradiated worms (P0) were transferred to NGM plates seeded with OP50 approximately 5 worms per plate, and were incubated at 20°C. 24 hours after exposure, P0 animals were transferred to fresh seeded plates to ensure that the first progeny of irradiated animals were not used. On the third and fourth day after UV exposure, 250 L4 animals from the F1 generation were picked to individual seeded plates, giving 500 F1 animals. On the seventh and eighth day after UV exposure, two F2 L4s from each F1 plate were transferred to individual plates, giving a total of 1000 F2s. The progeny of these F2 animals were screened for 100% transmission of the transgene, determined by expression of mRFP in the pharyngeal neurons. Following confirmation of ChR2 activity, integrated lines were backcrossed into N2 six times to remove background mutations. A single integrated transgenic line expressing *peat-4::ChR2;mRFP* was used in the microfluidic experiments.

ChR2 requires the cofactor retinal for light activation, thus prior to the experiment L4 worms were incubated overnight either off (control) or on retinal feeding plates as previously described [42].

Light illumination was achieved using a narrow bandwidth ultra bright blue (470 nM) LED (Maplin Electronics) as previously described [42]. The LED was placed on the top of the trapping channel in contact with the chip. Individual worms were loaded into the microfluidic chamber with 2 mM 5-HT to stimulate pharyngeal pumping. EPG signals were captured 2 minutes before, 2 minutes during and 2 minutes after LED illumination of the chamber.

## Results

### Capture of EPG Signals with NeuroChip

**Figure 3:**
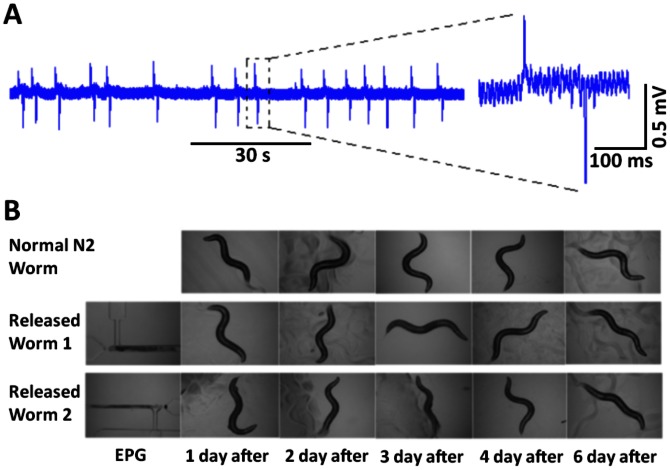
An example of an EPG recording collected from the tail and the recovery trapped and released worms. **A.** An electrophysiological recording made from the tail of a worm that entered the chamber in the wrong orientation. Note the lack of the features seen with the EPG waveform compared to Fig. 1C and the small amplitude of the signal. **B.** Worms can be recovered from the EPG chip following recording and show normal fecundity and viability. Wild-type (N2) worms that were not trapped in the device (top row, ‘normal’), or trapped and released (bottom two rows) were followed for development and survival over the course of six days. The worms that had been released from the device were alive, moved in a similar manner and produced the same number of progeny as untreated worms.

The pharynx of *C. elegans* consists of a radial muscle which is divided into three functional parts, the corpus near the mouth of the nematode, the isthmus (I) in the middle, and the terminal bulb (TB) (Fig. 2F) [30], [43], [44]. The integral neural circuit drives and regulates its activity. An EPG signal corresponds to a single pumping action from these three parts (a contraction followed by a relaxation) and a typical EPG signal comprises five phases, called e, E, P, R and r. These features can readily be resolved in a conventional microelectrode recording made from a cut head preparation of *C. elegans* (Fig. 1A) [27]; removing the head from the body of the worm improves the stability of the recording. These phases are generated with each complete pharyngeal muscle contraction relaxation cycle of wild-type worms. The excitation phase that drives muscle contraction consists of two positive spikes, a small one ‘e’ which derives from activity of the cholinergic pacemaker neurone MC [45] followed by a bigger one ‘E’ which records the electrical activity that underpins contraction of the pharynx [27]. The plateau phase contains several negative spikes ‘P’ which correlate with activity of the M3 inhibitory glutamatergic motor neurons [30]. The ‘R’ or relaxation phase typically comprises two negative transients, a bigger one ‘R’ resulting from synchronous repolarisation of the pharyngeal corpus and anterior isthmus followed by a small one ‘r’ which is due to terminal bulb repolarisation [27]. ‘E’ and ‘R’ spikes are useful for studying muscle excitability, and ‘e’ and ‘P’ waves are useful for studying excitatory and inhibitory synaptic functions, respectively. The pattern of activity provides information about coordinated activity in the neural network [31]. The components of the wild-type EPG signal are shown in Fig. 1A. All the neural features of the EPG can be seen in the recordings obtained with the new device (Fig. 1C, far right trace) which we have therefore called NeuroChip. We made a direct comparison of parameters obtained from conventional microelectrode recordings and NeuroChip which indicate that the amplitude, pump duration and waveform (e, E, P, R, r) are comparable using the two experimental approaches (Table 1). NeuroChip also captured electrophysiological signals from the tail of the worm: These recordings had a frequency and duration indicating they were likely to originate from pharyngeal activity (Fig. 3A) and are similar to those previously described [33]. They lack the detail of the EPG recorded from the head. Thus whilst they may be used to examine gross drug effects on the rate of pharyngeal pumping [33] they cannot be used to resolve the activity of different neural components of the pharyngeal circuit.

**Table 1:** A summary of EPG parameters compared for NeuroChip and conventional microelectrode recordings.

	Conventional recording	NeuroChip
***Wild-type (N2) (n = 5)***		
*EPG amplitude (mV)*	4.780±1.006	4.410±0.051
*EPG frequency (s^−1^)*	0.060±0.016	0.075±0.019
*EPG duration (s)*	0.112±0.015	0.116±0.006
*Average number of P waves (pump^−1^)*	0.590±0.143	1.788±0.225***^1^
*‘e’ amplitude (mV)*	0.203±0.039	0.257±0.037
*R-E interval (s)*	28.973±6.863	24.658±5.407
*R-E ratio*	1.006±0.097	1.222±0.085
***Wild-type with 5-HT (10*** ** ***mM) (n = 10)***		
*EPG amplitude (mV)*	7.580±0.769	8.370±0.835
*EPG frequency (s^−1^)*	1.878±0.120	3.467±0.104****^2^
*EPG duration (s)*	0.135±0.007	0.101±0.002***^3^
*Average number of P waves (pump^−1^)*	0.201±0.087	0.126±0.035
*‘e’ amplitude (mV)*	1.080±0.062****^4^	1.753±0.079****^5^
*R-E interval (s)*	0.550±0.170	0.198±0.009
*R-E ratio*	1.046±0.043	1.143±0.020

These recordings were made from the same populations of wild-type N2 worms. Data are the mean ± s.e.mean of recordings from ‘n’ worms. Each parameter was derived from either AutoEPG analysis of all the EPGs captured in a recording of 5 minutes or, for manual analysis, from 60 EPGs for ‘n’ worms. Comparisons made by unpaired Student’s t-test to the same parameter for ^1^conventional wild-type N2 recording without 5-HT (P = 0.001);

2 conventional wild-type N2 recording with 5-HT (P<0.0001);

3 conventional wild-type N2 recording with 5-HT (P = 0.0002);

4 conventional wild-type N2 recording without 5-HT (P<0.0001);

5 NeuroChip recording without 5-HT (P<0.0001).

### Worms can be Recovered and Propagated after NeuroChip Recording

To evaluate whether or not the trapping of the worms in the microfluidic device caused damage we conducted recovery experiments in which worms were captured in the channel, EPGs recorded for five minutes, and the worms then retrieved and placed back on culture plates. We measured the impact of trapping on motility by transferring individual worms onto an agar plate without food and after one hour counting the number of body bends made in 3 minutes. Control worms were treated in the same way except they were not trapped in the device prior to transfer to the agar plate. Trapped and released worms moved with a frequency of 21.3±6.4 body bends min^−1^ whilst controls moved at a frequency of 26.0±7.2 body bends min^−1^ (n = 3; mean ± s.d.). Thus trapping caused a small, approximately 20% reduction in motility. To test whether trapping impacted on a worm’s ability to grow and reproduce we released trapped worms onto individual culture plates with food and counted the number of progeny from each worm. Worms that had been released from the device were still alive and had a similar growth rate and propagation compared to un-trapped (‘normal’) worms (Fig. 3B). (Two days following release from the trap the average brood size for six worms was 104±4; mean ± s.d. This is similar to the expected brood size for 2 day old worms [46]). Overall, therefore, despite a slight reduction in motility, the trapped and released worms were able to grow and produce viable progeny.

Thus individual worms may be recovered and propagated following electrophysiological analysis with NeuroChip, an important consideration for applying this approach for mutant screens.

### NeuroChip Resolves EPG Signals that are Comparable to Conventional Recordings

**Figure 4:**
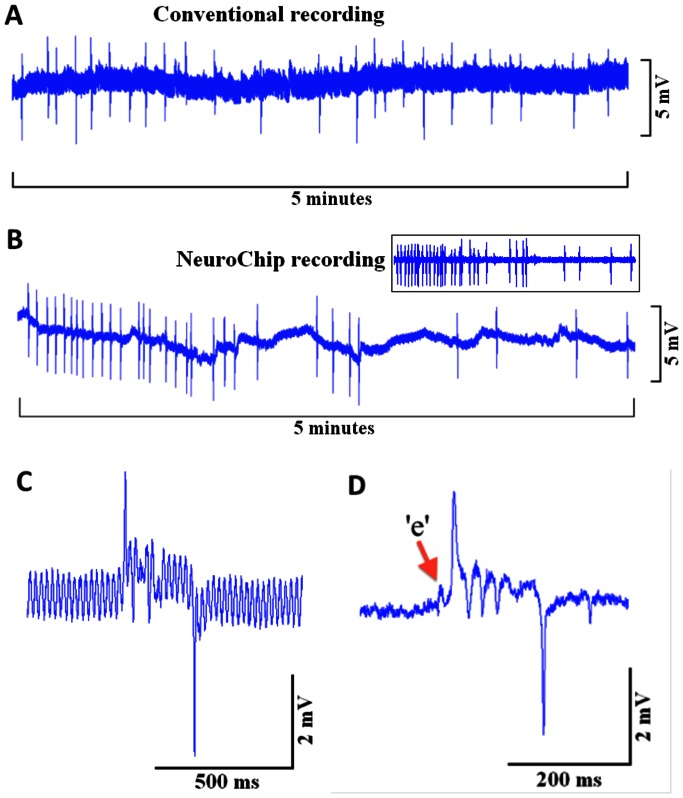
A comparison of basal EPG recordings with conventional microelectrodes and the microfluidic device. Recordings were made from wild-type worms in the absence of 5-HT and therefore the frequency of the EPGs is low (around 0.1 Hz). **A.** 5 minutes of conventional microelectrode recording from intact worm **B.** 5 minutes of recording using the NeuroChip. Drift in the baseline is probably likely due to residual movement of the worm in the chamber and can be subtracted with filtering if desired (inset shows signal filtered to remove this drift). Stable EPG waveforms can be captured for extended periods of time. **C.** An example of a single EPG waveform recorded using the conventional microelectrode. **D**. An example of a single EPG waveform recorded using the NeuroChip. Note the much improved signal to noise ratio in the NeuroChip compared with a conventional recording. This is due to the fact that often greater suction has to be exerted to secure the freely moving worm on the microelectrode.

**Figure 5:**
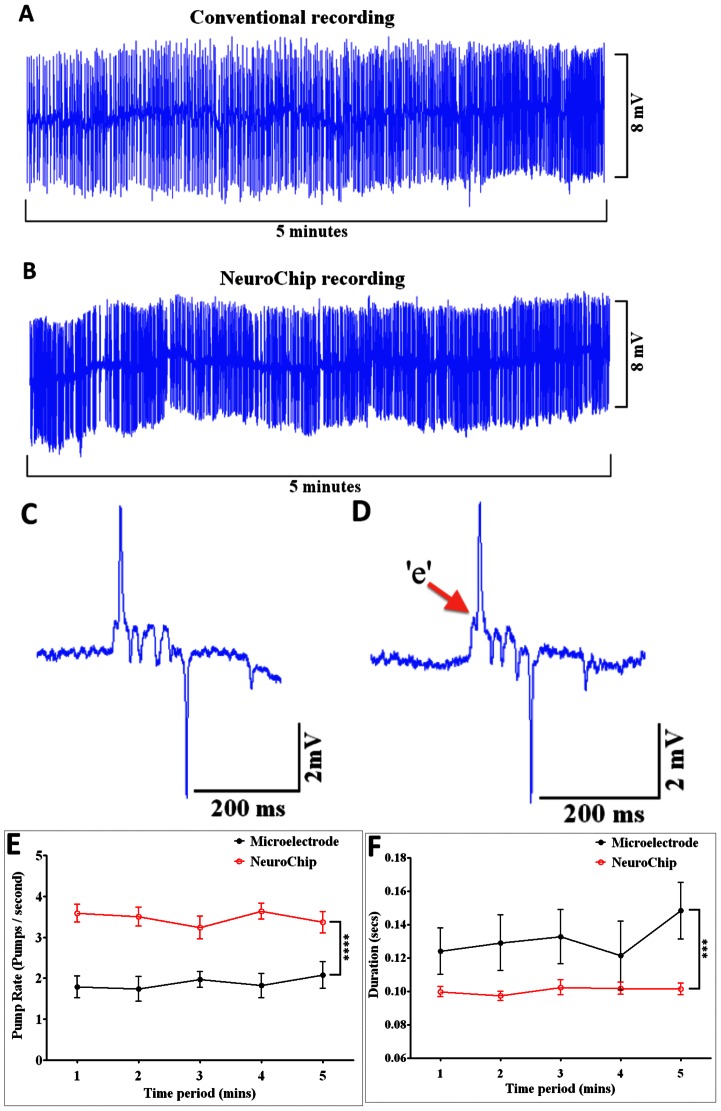
5-HT (serotonin) stimulated worms: comparison of conventional microelectrodes and the microfluidic device. Wild-type worms that were incubated in 10 mM 5-HT for at least 10 minutes before, and throughout the entire experiment. **A.** 5 minute trace using a conventional microelectrode **B.** 5 minutes trace using the NeuroChip. **C.** Example of a single EPG waveform recorded using the conventional microelectrode. **D.** Example of a single EPG waveform recorded using the NeuroChip. **E** and **F**. Time courses of the EPG frequency and EPG duration, comparing the stability of recording with conventional microelectrode or the NeuroChip. Data are the mean ± SEM. A higher frequency and shorter pump duration is observed with NeuroChip compared to conventional recordings, (n = 10). Note that conventional recordings from intact worms have an improved signal to noise ratio in the presence of 5-HT compared to without 5-HT, Fig. 3 C; Fig. 4C. 5-HT reduces motility [68], [69] and so less suction has to be applied to hold the worms in the recording electrode. Reduced seal resistance is predicted to reduce the noise [33].

In order to validate the utility of the microfluidic device in comparison to conventional microelectrode recordings, in particular with respect to resolving discrete neural components of the waveform, parallel experiments were performed using both methods on wild-type worms without 5-HT (Fig. 4) and with 5-HT (Fig. 5; Table 1). 5-HT (10 mM) stimulates pharyngeal pumping [47]–[49] and this was observed for both conventional and NeuroChip recordings. However, in the presence of 5-HT, the pump rate was greater and the pump duration was less for the NeuroChip than for conventional recordings (Table 1). An explanation for this is provided by a recent paper showing that the pharyngeal pumping rate of worms moving in liquid is inhibited [50]: Worms in the conventional recording configuration are still able to move their bodies vigorously, unlike worms trapped in the NeuroChip thus these latter worms might be expected to pump at a higher rate. As pharyngeal pumping frequency increases the pump duration typically decreases [49]. This also provides an explanation for the slightly lower pump duration observed with the device. An alternative possibility is that the pressure exerted to hold the worm in the trapping chamber may affect pharyngeal pumping in the NeuroChip device.

### Detection of Effects on Synaptic Cholinergic Signalling

Previous studies have shown that 5-HT increases signalling from the cholinergic pacemaker neurone MC [28], [49], [51] and this therefore predicts that 5-HT should increase the amplitude of ‘e’. NeuroChip is able to directly resolve this effect at least as reliably as conventional microelectrode recordings (Table 1; compare Fig. 4D with Fig. 5D) and therefore is capable of defining effects on excitatory cholinergic synaptic transmission. A caveat to this is that the amplitude of ‘e’ may be affected by the interval between ‘e’ and the larger ‘E’ spike. If this interval becomes briefer then ‘e’ may become superimposed on the ‘E’ spike which would contribute to an increase in amplitude as measured with the current approach. Future analyses could be optimised to resolve this e.g. by extracting the ‘e’ waveform from the ‘E’ spike.

### Rapid Application of Compounds and Simultaneous Electrophysiological Recording

**Figure 6:**
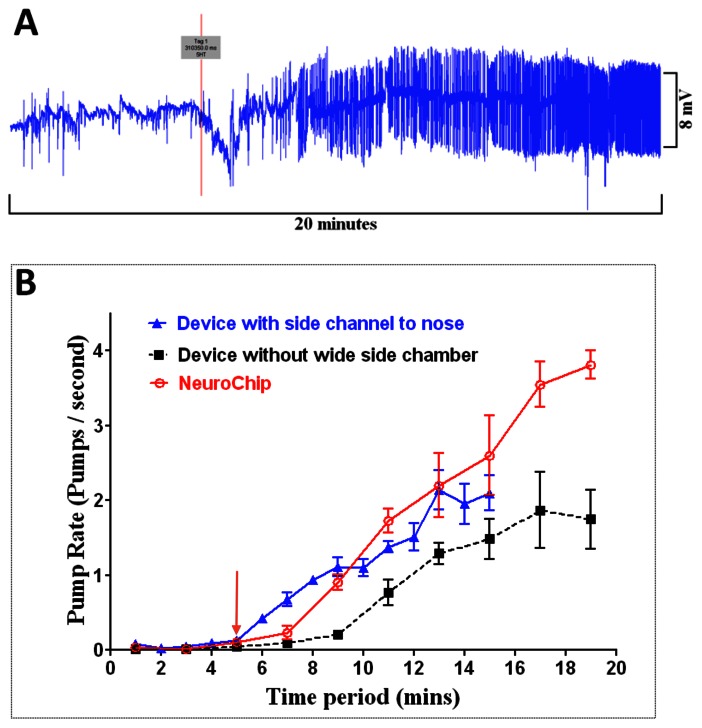
Optimising drug delivery time. NeuroChip recordings were made from wild-type worms whilst 5-HT (10 mM) was added via the drug port or outlet port (see Fig. 2C). Two different designs of device were tested. One with (NeuroChip) and one without a wide perforated chamber that juxtaposed the trapping channel (see Fig. 2G). **A.** EPG recording taken during exposure to the 5-HT. The red line indicates when the 5-HT was added to the drug port under a positive pressure of 1 bar. This recording was made using the device with the wide side chamber i.e. NeuroChip **B**. Response time to 5-HT for NeuroChip. The red arrow indicates when 5-HT was applied either via the drug port or via the outlet port as indicated. The dotted black line indicates the response time for the device without the wide side chamber. The blue line indicates the response time for NeuroChip with 5-HT added directly to the nose via the outlet port. The response to 5-HT in NeuroChip was significantly greater than the response that was recorded compared to the device without the side chamber (n = 5 worms for each design; data are mean ± SEM; ** P = 0.0089, Student’s t-test for the last time point). The maximum pump rate following 5-HT application to NeuroChip closely matched that observed in worms that were pre-incubated in 5-HT prior to addition to the device (see Table 1) suggesting full equilibriation of the drug with the worm. The experiments in which the 5-HT was added to the nose via the outlet port had to be terminated after 15 minutes as the increased pressure in the channel made it difficult to maintain the worm in the trap. However, 2 minutes after addition of 5-HT there was a significantly greater increase in frequency when 5-HT was applied to the nose compared to addition via the drug port (n = 5 worms for each design P = 0.0013, one-way ANOVA) suggesting that the onset of response might be slightly faster following this route of application.

For drug screening and chemical biology it is advantageous to have the capability to rapidly apply and remove drugs or chemicals whilst simultaneously capturing the EPG waveform to monitor the effect on the activity of the neural circuit. Dye experiments indicate that drug access to the channel of NeuroChip is very rapid (Video S2). Using NeuroChip we observed responses to 5-HT that had a latency of 5 min and achieved the maximal effect (i.e. same pump rate as worms that were pre-exposed to 5-HT before addition to the chamber) after 15 minutes (Fig. 6 A, B). The time taken for the full response to 5-HT to develop likely reflects the time taken for 5-HT to get access to its site of action inside the worm. We considered that this might be improved if the device was configured to allow drug to flow across the mouth of the worm. However, when 5-HT was applied directly to the mouth of the worm there was no significant overall effect on the time-course of the response (Fig. 6B).

**Figure 7:**
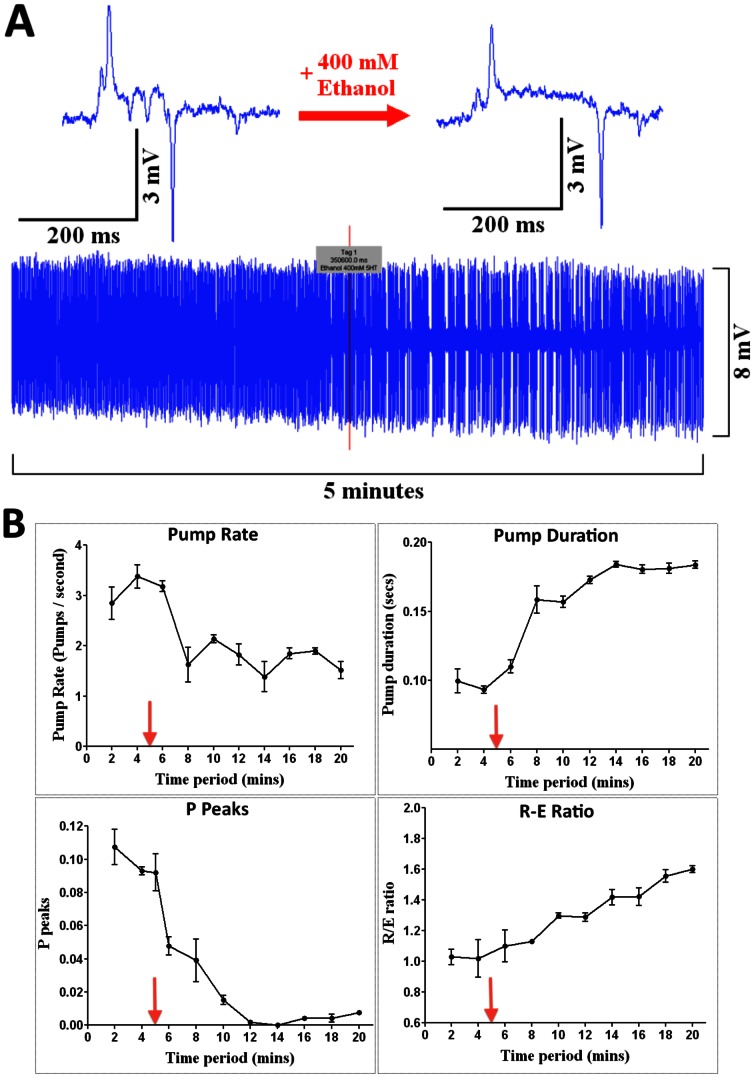
Detecting modulatory drug effects with NeuroChip. Wild-type worms in the presence of 10 mM 5-HT were used. Ethanol was applied through the drug port of NeuroChip. **A**. An example of the change in the EPG waveform with 400 mM ethanol and (below) a 5 minute recording showing the time-course of the ethanol effect. The red line indicates the point at which ethanol was added to the drug port. Note the rapid reduction in EPG frequency. **B.** Ethanol modulates four parameters of the EPG (red arrows indicate the time of addition of ethanol via the drug port); n = 3 worms for each graph; data are mean ± SEM.

We also tested the response time to compounds for which there is evidence that the cuticle does not present a barrier i.e. ethanol [40] and for which the rate-limiting factor in the response time would be the time taken for the drug to get access to the recording chamber. For these experiments the response time was very rapid, t_1/2_<2 min, (Fig. 7) supporting the contention that the cuticle does not present a significant diffusion barrier to ethanol [40]. Ethanol (400 mM) had a modulatory effect on 5-HT (10 mM) stimulated pharyngeal pumping causing a decrease in frequency, an increase in pump duration, a decrease in the number of ‘P’ waves per pump and a relative reduction in the amplitude of the ‘E’ spike (Fig. 7).

### Detection of Effects on Inhibitory Neurotransmission and Mutant Sorting

We tested whether or not the microfluidic device could detect changes in inhibitory neurotransmission. The *eat-4* mutant was chosen for this study as it harbours a loss of function mutation in a vesicular glutamate transporter and is deficient in inhibitory glutamatergic signalling [29]. In the pharyngeal system this leads to loss of signalling through the inhibitory glutamatergic motorneurone M3 with a concomitant and distinctive loss of the EPG ‘P’ waves, which result from M3 activity, and a characteristic lengthening of pump duration [29]. Similar to the comparison made for wild-type worms (Table 1), for *eat-4* the pump frequency was higher and duration shorter in NeuroChip compared to conventional recordings, again perhaps reflecting the impact of constraining worm movement in the microfluidic chamber on pumping rate [50]. The number of ‘P’ waves per pump and the R/E ratio was different between conventional and NeuroChip recordings for *eat-4* (Table 2) but the significance of these differences are unclear. Importantly, a comparison of the data obtained from the mutant *eat-4* (*n2474*) (Table 2) with that of wild-type (Table 1) confirmed the capability of NeuroChip to detect changes in inhibitory neural transmission. As with conventional recordings, and consistent with published data [29] *eat-4* was shown to have a reduced number of ‘P’ waves and increased pump duration (Table 1; Table 2).

**Table 2:** *eat-4* mutants recorded with NeuroChip or conventional microelectrode recordings have the same phenotype.

	Conventional recording	NeuroChip
***Eat-4(n2474)*** ** (n = 5)**		
EPG frequency (s^−1^)	0.055±0.007	0.090±0.015
EPG duration (s)	0.287±0.014****^1^	0.172±0.009****^3^
Average number of P waves	0.280±0.065*^2^	0.103±0.022****^4^
‘e’ amplitude (mV)	0.368±0.030	0.376±0.034
R-E interval (s)	26.513±3.078	22.288±3.867
R-E ratio	2.170±0.117	1.491±0.085

Data are the mean ± s.e.mean of recordings from ‘n’ worms. Each parameter was derived from AutoEPG analysis of all the EPGs captured in a recording of 5 minutes or, for manual analysis, from 60 EPGs. *Eat-4* had increased pump duration and decreased number of ‘P’ waves compared to wild-type. Comparisons made by unpaired Student’s t-test to the respective parameters for ^1,2^ conventional wild-type N2 recording without 5-HT, P<0.0001 and P = 0.0208, respectively; or ^3,4^ to wild-type N2 NeuroChip recording without 5-HT^,^ P<0.0001. Wild-type data are provided in Table 1.

**Figure 8:**
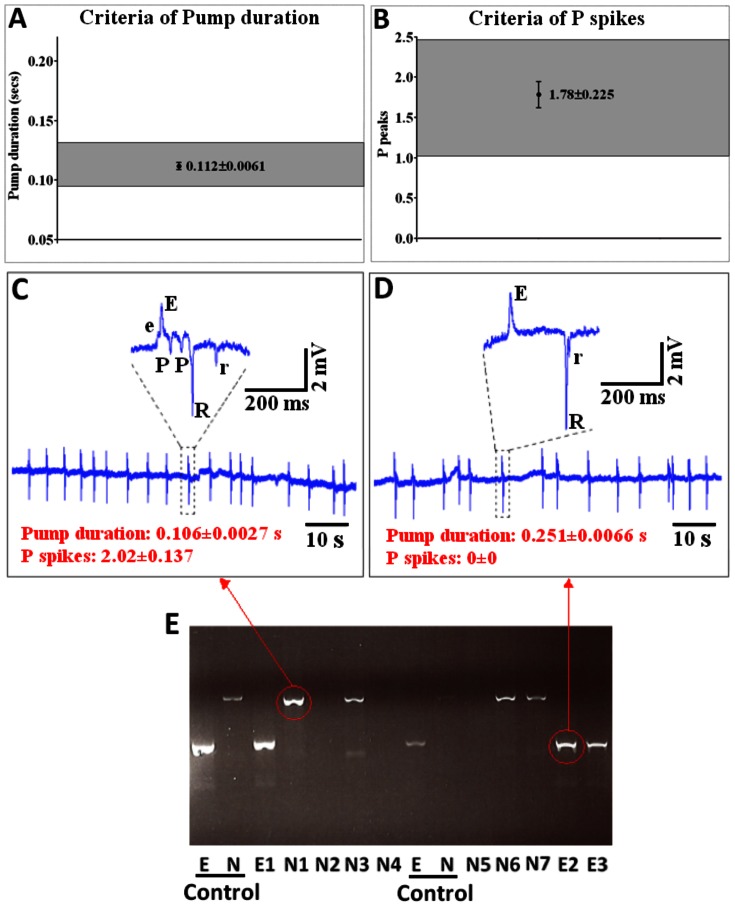
NeuroChip detection and selection of *C. elegans* mutants. A population of wild-type worms was spiked the mutant *eat-4* (7 N2 wild-type plus 3 *eat-4*) and subjected to NeuroChip analysis to select the mutant worms. **A.** Criteria of EPG pump duration and **B.** number of ‘P’ waves per pump that were used for the selection of mutant from wild-type worms. The boundaries of the wild-type phenotype were defined as three times the standard deviation of the mean for wild-type recordings. Any worm with a mean value lying outside this boundary was identified as a mutant. **C**. Example of an EPG recording obtained from wild-type worm and **D.** from the mutant *eat-4(ky5)*. Note the longer pump duration and absence of P waves. **E.** Matching the NeuroChip phenotype to the worm genotype by PCR. Genotyping was by PCR to amplify the *eat-4* locus and confirmed whether or not the worm carried the genomic deletion. ‘E’ and ‘N’ are the reference lanes for *eat-4* and wild type, respectively. N1– N7 are worms that were sorted as wild-type and E1–E3 are worms that were sorted as mutants from the NeuroChip, phenotyped as *eat-4*. In three of the samples identified as wild-type the PCR reaction failed (N2, N4 and N5), but in the remaining samples the PCR result confirms the phenotype assigned by the NeuroChip analysis. There were no false positive or false negative results. The red arrows indicate the corresponding PCR result for the two EPG traces shown in C and D.

Genetic screens of *C. elegans* pharyngeal phenotypes by visually scoring the activity of the pharynx in intact worms has been of great utility in identifying novel mutants and providing insight into gene function [52]. We therefore tested whether or not NeuroChip provides an opportunity to resolve mutant from wild-type pharyngeal function. For this we sorted mutant *eat-4* worms from wild-type on the basis of electrophysiological signature. A wild-type population of worms was spiked with mutant worms, *eat-4(ky5)*, and a sample was analysed on NeuroChip. EPG signals were collected from worms loaded from a reservoir and analysed in real time. For a worm to be identified as ‘wild-type’ it had to fulfil the criteria of having a pump duration and number of ‘P’ waves per pump within three times the standard deviation of the mean established for wild-type recordings in the validation experiment (Fig. 8A and B). Thus any worm that had average pump duration greater than 0.1445 s and number of ‘P’ waves less than 0.169 was identified as a mutant i.e. *eat-4* (Fig. 8C and D). On these criteria three worms were identified as *eat-4*. Subsequent genotyping by single worm PCR confirmed that all three worms that were identified as *eat-4* by NeuroChip carried the *eat-4* mutation i.e. there were no false positives (Fig. 8E). Thus, NeuroChip is capable of mutant sorting from wild-type worms based on the electrophysiological signature. To test whether or not there were false negatives in a NeuroChip mutant screen we carried out a further analysis using a strain carrying both the *eat-4 (ky5)* mutation and red fluorescent protein, *eat-4(ky5);peat-4::ChR2;mRFP*. This permitted robust discrimination of wild-type worms from *eat-4* worms after mutant selection and screening on the basis of the absence of red fluorescence. For this experiment 18 wild-type and 13 *eat-4* mutants were mixed together and then 20 were randomly transferred to the loading chamber. 17 worms were recorded head-first in the correct orientation for EPG and of these 8 were identified as wild-type and 4 as *eat-4* on the basis of EPG signal. Subsequent visual inspection (independent observer, single blind trial) confirmed this identification as correct. Thus this screen generated no false negatives. However, a caveat to this is that the current device cannot phenotype worms that enter the device tail first. A solution to this would be to carry out an iterative approach in which worms that entered tail first were collected and re-applied to the device.

### Throughput

When conducting the genetic screen described above we timed the throughput. In 1 hour 2 min, 15 worms were recorded (total time taken, from loading reservoir and adding first worm to the channel to release of the last worm). Of these, three were tail first and discarded; thus 12 EPGs were recorded in this time period. The rate-limiting step is the time required to record enough EPG waveforms in order to delineate a wild-type or mutant signature, up to 5 minutes depending on the pumping frequency. This experiment demonstrates an initial capability of a throughput of 12 worms hour^−1^. This is a significant improvement on the conventional microelectrode recording approach which is slower because of the need to make the microelectrode, fill it, position it and manually capture the motile worm. A skilled operator employing the conventional approach would have a throughput maximum of about 2 to 4 recordings hour^−1^.

### Interrogating Neural Network Properties with Optogenetics

**Figure 9:**
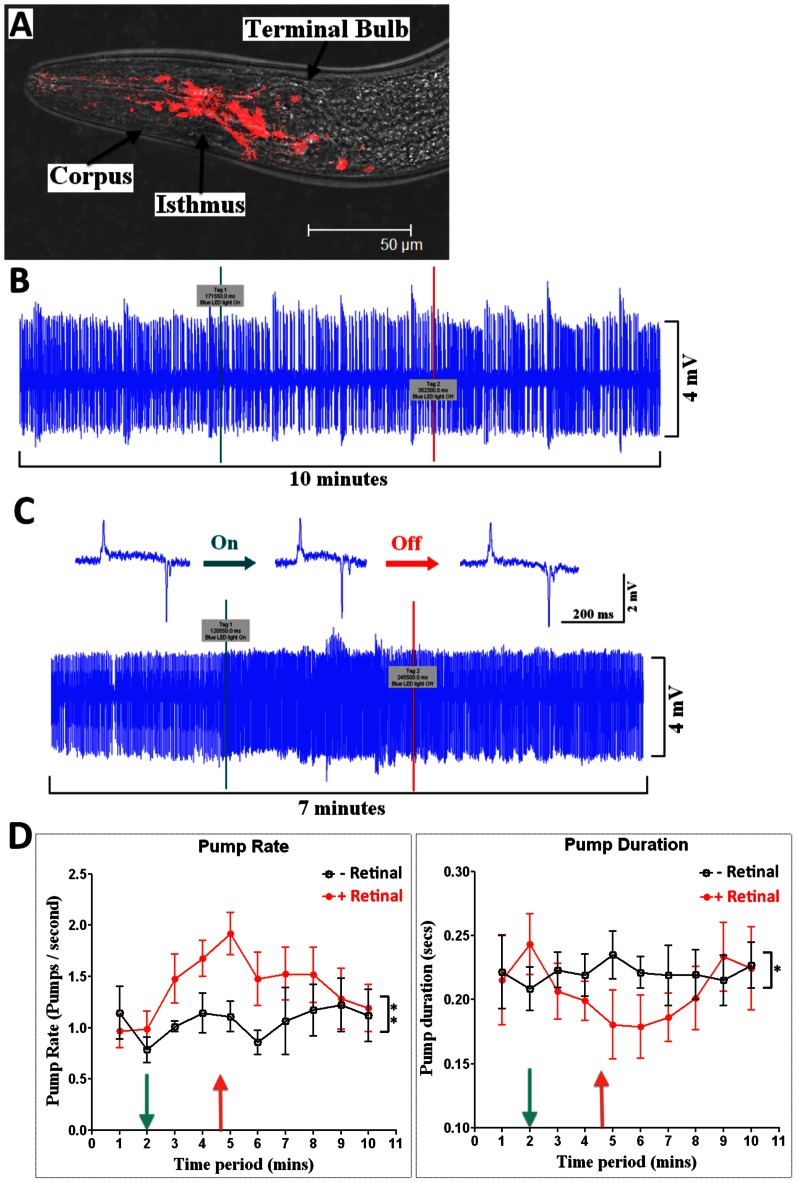
Interrogation of neural network properties with optogenetics. A *C. elegans* strain expressing the blue-light activated ion channel (470 nm) ChR2 in glutamate neurones was used (*peat-4::ChR2;mRFP*). **A.** Confocal image of an adult worm (L4+1 day; *peat-4::ChR-2;mRFP*). Red fluorescence reports the expression pattern of the light-activated channel ChR2 and shows expression in glutamate neurones of the nervous system. NeuroChip recordings were made from these worms in the presence of 2 mM 5-HT. Control recordings were made in worms that had not been pre-treated with retinal, the cofactor for ChR2 activation. **B.** Illumination (470 nm) of worms that had not been treated with retinal had no effect on EPG recordings. Light on at 3 min (green line) and off at 6 min (red line). **C.** EPG recording obtained from a retinal treated worm. Light on at 2 min (green line) and off at 4.5 min (red line). LED illumination increased the pump rate and decreased the pump duration, which can be observed from single EPG traces (shown above). **D.** Pump rate and duration in response to light activation with (red curve) and without (black curve) retinal. Data are the mean ± SEM; n = 4. Pump rate was significantly increased following light activation in the retinal treatment group (P<0.001; paired Student’s t-test, 2 min compared to 6 min) and overall pump rate was higher in the retinal treatment group compared to control during light activation (two way ANOVA; F = 8.03 P = 0.0298 for the retinal treated group compared to control during light activation). Light activation significantly decreased pump duration in the retinal treatment group (P<0.01 paired Student’s t-test, 2 min compared to 6 min) whilst there was no change in the control group (P = 0.2261 paired Student’s t-test, 2 min compared to 6 min).

Optogenetics is a powerful approach for interrogating the function of neural networks. It deploys genetically encoded light sensitive ion channels to provide a means of remotely activating specific neural pathways by light [53] and has previously been used to activate neural pathways in *C. elegans* [54] including in the pharyngeal nervous system [42]. Here we tested whether NeuroChip could resolve optogenetic modulation of the neural network mediated via glutamatergic neurone activation. For this we used a transgenic strain of *C. elegans* stably expressing Channelrhodopsin2 (ChR2) exclusively in glutamate neurones (*peat-4::ChR2;mRFP*). Worms treated with 5-HT were subjected to light (470 nm) illumination whilst capturing EPG signals. Control experiments were conducted in parallel in which the worms were not treated with retinal, the co-factor for ChR2 activation. A selective light-dependent decrease in pump duration was observed in retinal treated worms but not in controls (Fig. 9). This recapitulates an earlier study which used a photolysable ‘caged’ glutamate compound and observed a light-dependent decrease in pump duration [30]. The optogenetic approach in combination with the NeuroChip permitted capture of EPG signals over a long time course and thus also resolved a concomitant and sustained increase in pharyngeal frequency following light activation of *peat-4;ChR2;mRFP* (Fig. 9D). A neurobiological explanation of this observation can be provided by previous studies which have demonstrated a key role for glutamate signalling in shortening the duration of the pharyngeal contraction-relaxation cycle in the presence of 5-HT [49]. This suggests that optogenetic activation of glutamate signalling in the presence of 5-HT may act to shorten the contraction-relaxation cycle thus permitting the pharynx to pump at a faster rate. An alternative or additional explanation is that glutamate may be directly excitatory to the pharynx and this is supported by observations of glutamate-dependent depolarisation of pharyngeal muscle from intracellular recordings [55].

### NeuroChip can be Modified for Smaller Nematodes

**Figure 10:**
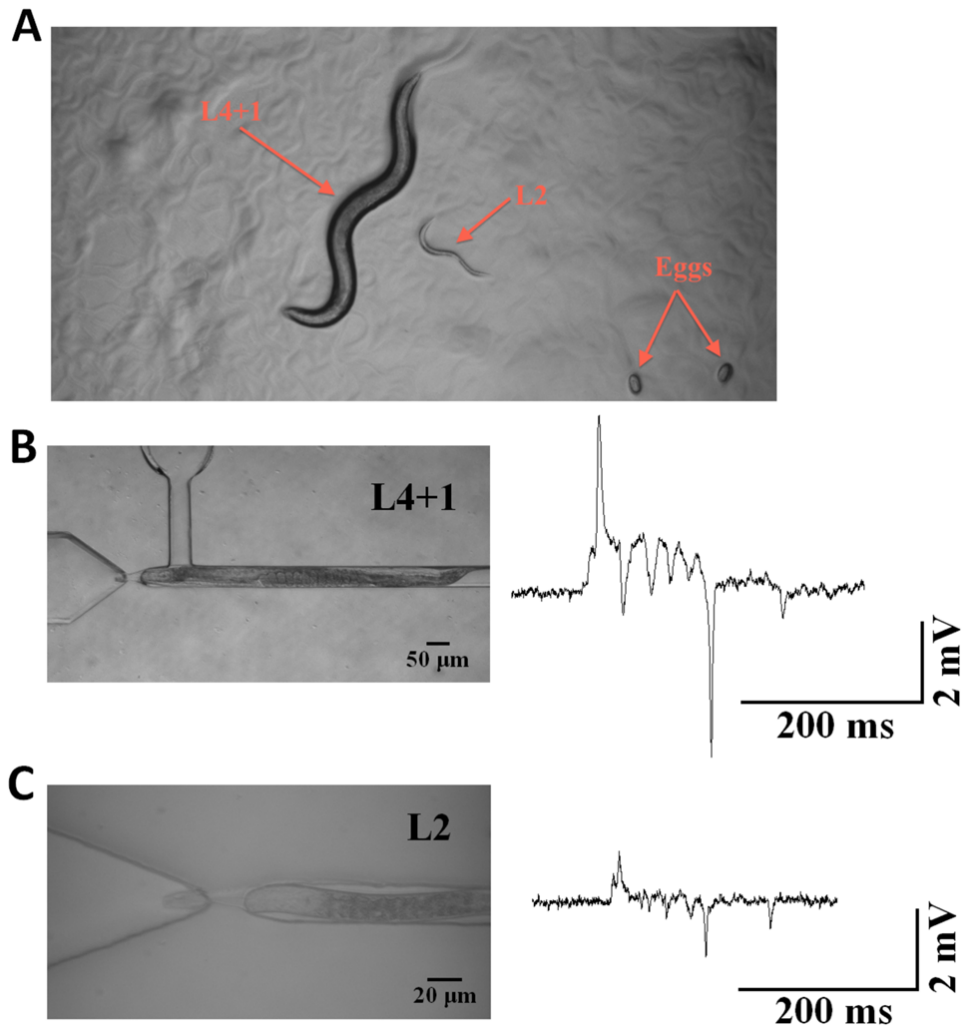
NeuroChip can be modified to record from smaller size worms. The aperture of the trapping channel was decreased to accommodate L2 larva stage *C. elegans* **A.** Comparison of the sizes between the adult (one day after L4 larva) and L2 larva. Adult worms are between 1110–1150 µm whilst L2 are 360–380 µm [56] **B.** Example of a trapped adult *C. elegans* and the recorded EPG signal. **C.** Example of a trapped L2 larva *C. elegans* and the EPG signal. Although the amplitude of the EPG is smaller, nonetheless each phase and feature can be resolved in the waveform.

Previous experimental investigations have required EPGs to be recorded from early developmental stages of *C. elegans* which are considerably smaller in size that adult worms [56]. Thus, after several iterations, we modified the trapping channel in NeuroChip to an aperture that could capture EPG signals from L2 *C. elegans* larvae (Fig. 10; Video S3). The waveform of these recordings was similar to those previously described for L1 larvae [56].

## Discussion

Electrophysiological recordings from neural circuits with suction or extracellular electrodes have the advantage that they can capture a waveform that reports on the activity of a number of neural components in any given circuit encompassing excitatory, inhibitory and modulatory signals [27], [57]–[60] and thus provide a broadly informative route to defining the effect of drugs or mutations on neural function. Extracellular recordings from the *C. elegans* pharyngeal network have been useful in this regard [61] but *C. elegans* is a fast-moving nematode and therefore it is not easy to collect EPG recordings from the whole nematode with the conventional suction electrode approach [27]. First, it is hard to trap the worm’s head when it is vigorously swimming. Second, once the worm is trapped, more suction is required to keep it in position leading to a noisier signal which requires filtering. Cut head preparations may be used to overcome this problem but this is more time-consuming and technically challenging and not amenable to a high throughput approach. Thus approaches have been developed to circumvent this by the design of a microfluidic chamber for intact worm EPG recordings that completely replaces the need for micromanipulation and a conventional extracellular microelectrode [32]–[34]. The microelectrode function is performed by a trapping chamber, which acts in a similar fashion to classic sucrose-gap recordings [57]. Here we show that by changing the shape of the aperture from a square aperture to an opening that moulds to the shape of the worm’s head, thus more closely mimicking the way the worm is held in conventional microelectrode recording, it is possible to capture a detailed waveform which can reliably detect components of the EPG which report neuronal activity. This represents an important development in this experimental approach as it opens the way for a precise definition of neuroactive compounds and mutations with synaptic phenotypes. The properties of the EPG waveform recorded during basal activity with either the conventional microelectrode or with NeuroChip were very similar with the exception that the device resolved more ‘P’ waves per pharyngeal contraction-relaxation cycle, or ‘pump’ (Table 1). Recordings were also highly reproducible between worms conferring advantage for the detection of discrete electrophysiological phenotypes.

The reproducibility of the EPG recording is an important feature of NeuroChip which we demonstrate confers capability for sorting worms on the basis of electrophysiological phenotype. This indicates applicability for mutagenesis screens with the potential to deliver new biological insight into neural signalling and synaptic function as has recently been achieved for other imaging based screens employing *C. elegans* e.g. [25]. We show that NeuroChip can be used for selecting mutant worms from a wild-type population and for recovering viable worms following recordings, both of which are important properties if this platform is to be utilised for mutagenesis screens. In this regard the integration of NeuroChip with a platform for optogenetics adds the capability to stimulate and record responses due to activation of discrete components of the neural network and further extends its capability for neurogenetic investigation.

The activity of the *C. elegans* pharyngeal circuit is regulated by a plethora of neurotransmitter receptors and channels which represent major targets for human medicines and anthelmintic drugs and through which toxic drug side effects may also occur [62]. Indeed, the *C. elegans* pharyngeal preparation has proven a useful system for characterising the mode of action of anthelmintic drugs such as ivermectin and emodepside [55], [63]–[65]. Thus NeuroChip has applications in chemical biology and toxicology screens. It incorporates a design feature which permits very rapid drug application thus optimising the device for such chemical screens. The throughput of the single channel device is 12 worms hour^−1^ (all in the correct orientation for EPG recording). Currently operation of the device is optimal with visual inspection of the worm in the trap so that worms entering tail first may be identified and future refinement will seek to automate this aspect. Furthermore, as Lockery et al demonstrate [33] it is possible to incorporate at least 8 channels on each microfluidic chip and thus the throughput could readily be increased to ∼100 worms per hour^−1^.

The size of the NeuroChip trapping channel and its opening dictates the size of the worm that can be captured and recorded. We have demonstrated that the design can readily be modified to accommodate L2 larval stages of *C. elegans*. There are very few reports of EPG recordings from larval stages, presumably reflecting the technical challenge of this in the small, highly motile worms [66]. Thus NeuroChip opens the way for a precise characterisation of the pharyngeal network in the developing organism. Furthermore, the dimensions of L2 *C. elegans* larvae are in the same order as many species of nematode of interest because of their relevance to human and animal disease and crop damage. Thus, by demonstrating the utility of NeuroChip for smaller size worms we show its potential for recordings from these parasitic species of medical and economic importance.

NeuroChip also has application in pharmacokinetic studies as it provides the opportunity to precisely capture the time-course of drug response and the ability to make comparisons on different genetic backgrounds which have modified susceptibility to drugs or chemicals e.g. drug transporter mutants. Such insight is useful particularly with respect to the emergence of drug resistance in parasitic nematodes the mechanisms of which may involve mutations affecting drug transport [67].

There is scope to further increase the throughput by fully automating the data capture and online extraction of EPG parameters. In conclusion, NeuroChip delivers a new tool for sorting worms on the basis of electrophysiological phenotype with applications in neurotoxicology, drug discovery and neurogenetics.

## Supporting Information

Video S1 **Rate of access of dye to the prototype device. Dye was added to the drug port at the time indicated in the video. This device did not have the wide perforated side chamber.**
(MP4)

Video S2 **Rate of access of dye to NeuroChip. Dye was added to the drug port at the time indicated in the video.**
(MP4)

Video S3 **Video of trapped L2** ***C. elegans*** **and accompanying, unfiltered, EPG signal.**
(WMV)
